# The Scientific Rationale for the Introduction of Renalase in the Concept of Cardiac Fibrosis

**DOI:** 10.3389/fcvm.2022.845878

**Published:** 2022-05-31

**Authors:** Dijana Stojanovic, Valentina Mitic, Miodrag Stojanovic, Jelena Milenkovic, Aleksandra Ignjatovic, Maja Milojkovic

**Affiliations:** ^1^Institute of Pathophysiology, Faculty of Medicine, University of Niš, Niš, Serbia; ^2^Department of Cardiovascular Rehabilitation, Institute for Treatment and Rehabilitation “Niska Banja”, Niska Banja, Serbia; ^3^Department of Medical Statistics and Informatics, Faculty of Medicine, University of Niš, Niš, Serbia; ^4^Center of Informatics and Biostatistics in Healthcare, Institute for Public Health, Niš, Serbia

**Keywords:** renalase, cardiac fibrosis, chronic inflammation, ERK 1/2, MAPK, sirtuins, EMT

## Abstract

Cardiac fibrosis represents a redundant accumulation of extracellular matrix proteins, resulting from a cascade of pathophysiological events involved in an ineffective healing response, that eventually leads to heart failure. The pathophysiology of cardiac fibrosis involves various cellular effectors (neutrophils, macrophages, cardiomyocytes, fibroblasts), up-regulation of profibrotic mediators (cytokines, chemokines, and growth factors), and processes where epithelial and endothelial cells undergo mesenchymal transition. Activated fibroblasts and myofibroblasts are the central cellular effectors in cardiac fibrosis, serving as the main source of matrix proteins. The most effective anti-fibrotic strategy will have to incorporate the specific targeting of the diverse cells, pathways, and their cross-talk in the pathogenesis of cardiac fibroproliferation. Additionally, renalase, a novel protein secreted by the kidneys, is identified. Evidence demonstrates its cytoprotective properties, establishing it as a survival element in various organ injuries (heart, kidney, liver, intestines), and as a significant anti-fibrotic factor, owing to its, *in vitro* and *in vivo* demonstrated pleiotropy to alleviate inflammation, oxidative stress, apoptosis, necrosis, and fibrotic responses. Effective anti-fibrotic therapy may seek to exploit renalase’s compound effects such as: lessening of the inflammatory cell infiltrate (neutrophils and macrophages), and macrophage polarization (M1 to M2), a decrease in the proinflammatory cytokines/chemokines/reactive species/growth factor release (TNF-α, IL-6, MCP-1, MIP-2, ROS, TGF-β1), an increase in anti-apoptotic factors (Bcl2), and prevention of caspase activation, inflammasome silencing, sirtuins (1 and 3) activation, and mitochondrial protection, suppression of epithelial to mesenchymal transition, a decrease in the pro-fibrotic markers expression (’α-SMA, collagen I, and III, TIMP-1, and fibronectin), and interference with MAPKs signaling network, most likely as a coordinator of pro-fibrotic signals. This review provides the scientific rationale for renalase’s scrutiny regarding cardiac fibrosis, and there is great anticipation that these newly identified pathways are set to progress one step further. Although substantial progress has been made, indicating renalase’s therapeutic promise, more profound experimental work is required to resolve the accurate underlying mechanisms of renalase, concerning cardiac fibrosis, before any potential translation to clinical investigation.

## Introduction

Cardiac fibrosis is a foreseeable and sometimes likely response to myocardial injury, regardless of the underlying etiology, and is characterized by the net accumulation of extracellular matrix proteins in the cardiac interstitium (1–9). To date, owing to the complexity of the cell types and signaling networks involved, the meticulous pathophysiology of cardiac fibrosis remains one of the major challenges in modern cardiovascular medicine. Considering the 800,000 fibrosis-related deaths worldwide annually, mostly due to heart and lung complications (2), cardiac fibrosis has represented a burden on the healthcare system, calling for an immediate conjoining of all available scientific expertise. Despite its prevalence, there is a scarcity of effective approaches for the suppression or reversion of cardiac fibrosis (8), most likely because cardiac fibrosis does not represent a single disease, but rather a general inappropriate response to trauma (1).

Within a pathophysiological context, every cardiac injury, owing to the vague regenerative capacity of an adult heart, results in the formation of a collagen-based scar (1, 2, 7), which aims to maintain the structural and functional integrity of the myocardium, and critically prevent cardiac rupture (1, 2). The initial event underlying fibrotic feedback is cardiomyocyte death (such as acute myocardial infarction), that leads to inflammation and subsequent activation of the profibrotic signaling network, ultimately resulting in the activation of fibroblasts, and scar formation. In the setting of sustained or exaggerated reparative programming activation, extensive deposition of extracellular matrix proteins occurs, ultimately affecting the structure and function of the heart (1, 2, 7). Nevertheless, several other pathophysiologic conditions (even if the cardiomyocytes are preserved), such as systemic hypertension, aging, obesity, and diabetes induce interstitial and perivascular deposition of collagen, and promote myocardial interstitial fibrosis, potentially contributing to the pathogenesis of heart failure (1).

Additionally, renalase, a novel protein secreted by the kidneys, is identified. Mounting evidence demonstrates the cytoprotective properties of this innovative molecule, establishing it as a key survival element in various organ injuries, and tumors, in gestation, and, more importantly, as a significant anti-fibrotic factor (10–25). There has been a significant breakthrough over the past few years, and is currently in progress, in understanding renalase’s pathophysiology, and its experimentally (*in vivo* and *in vitro*) demonstrated pleiotropy to alleviate inflammation, oxidative stress, apoptosis, necrosis, and fibrotic responses. This research opens the door to potentially important therapeutic assessment, and supports the hypothesis for renalase to become a brand-new molecule included in anti-fibrotic strategies. Moreover, renalase’s pathophysiological relevance in the protection of various organs (e.g., heart, kidney, liver, intestines) implies that if an anti-fibrotic approach seeks maximum efficiency, it may likely benefit from such a multifaceted molecule.

The goal of this review is to comprehensively address the diverse mechanisms of renalase’s action within the context of cardiac fibrosis, concerning its anti-inflammatory, anti-oxidative, anti-apoptotic, and anti-fibrotic traits. It has to be underscored that this paper is not dedicated to the pathophysiology of cardiac fibrosis exclusively, nor is it proposing some new insights into fibrosis pathogenesis, but rather provides the scientific rationale for renalase’s scrutiny regarding cardiac fibrosis. Moreover, the compiled data herein may be used as an accurate reference for renalase’s pathways, so far identified, and may be of value for subsequent experimental research design regarding renalase’s emerging potential in the area of cardiac fibrosis research. Therefore, it may be hypothesized that renalase administration, at least to some extent, represents an approach for challenging the fibroproliferative drive after initial cardiac injury. To the extent of our knowledge, this review represents some original perspectives regarding renalase analysis in the context of cardiac fibrosis, and there is great anticipation that these newly identified pathways in the research of cardiac fibrosis are set to progress one step further. The initial discussion will emphasize key molecular mechanisms involved in cardiac fibrosis, and its potential relationships with renalase will be thoroughly discussed throughout this review.

## The Pathophysiology of Cardiac Fibrosis

Cardiac fibrosis is characterized by the expansion of the myocardial interstitium *via* extracellular matrix (ECM) proteins deposition (e.g., collagens), associated with the extensive range of cellular and signaling pathways involved in the fibrotic response (1–9, 26, 27), and is a common pathophysiologic process of many cardiovascular diseases (1, 26). In the majority of cases, cardiomyocyte death represents the initial stimuli for triggering a cascade of pro-fibrotic malaise, however, other pathophysiological stimuli, such as a pressure overload, diabetes, obesity, and aging, even if the cardiomyocytes are preserved, may also activate fibroproliferative response (1, 26). The evolution of cardiac fibrosis involves the following: various cellular effectors (e.g., neutrophils, macrophages, mast cells, lymphocytes, cardiomyocytes, pericytes, epicardial epithelial cells, and vascular endothelial cells), whose functions are mostly context-dependent, activated at various time intervals; stimulation of profibrotic mediators, such as the angiotensin/aldosterone axis, proinflammatory cytokines, chemokines, and growth factors (e.g., transforming growth factor-β, TGF-β, and platelet-derived growth factors, PGDF); and processes where epicardial epithelial cells and endothelial cells undergo mesenchymal transition (1–9, 26–41). However, the up-regulation of these actions suggests acquiring a myofibroblastic phenotype of the quiescent fibroblasts, presented with secretory and contractile traits, and serving as the main source for extracellular matrix (ECM) proteins (1, 3, 5–7, 26–41).

Nevertheless, after an initial injury, myocardial tissue is transformed into a hostile and unsteady framework with a plethora of necrotic and apoptotic cardiomyocytes with immune cell expansion, followed by fibroblast activation, and proliferation (1, 3, 5–7, 26). These inflammatory cells initially accumulate near the lesion to resolve the injury, and facilitate healing, which remarkably alters the cardiac microenvironment (1, 3, 5–7, 26), thus providing a setting for the onset of fibrosis. In this respect, neutrophils represent the first cells recruited to the site of cardiac injury (3, 5, 6, 26, 29, 30), in order to release granular content (e.g., myeloperoxidase, cathepsins, and neutrophil gelatinase associated lipocalin), as well as expressing its phagocytic nature, providing extracellular chromatin traps which have been enriched with inflammatory enzymes (5). In addition to being quintessential proinflammatory cells, substantial evidence indicates that neutrophils serve as introductory modulators of the cardiac healing and remodeling process (3, 5, 26, 29), by means of phenotype shifting (N1 to N2) (30). Accordingly, neutrophils generate several pro-fibrotic mediators, namely interleukin (IL)-1β, NADPH oxidase 4 (Nox4), and metalloproteinases (MMPs), that, in conjunction with large amounts of generated reactive oxygen species (ROS), induce cardiac fibroblasts transdifferentiation, *via* protein kinase B/mammalian target of rapamycin (Akt/mTOR) nuclear factor-κβ (NF-κβ) signaling (3). Moreover, neutrophils, upon the onset of nod-like receptor protein (NLRP)3 inflammasome activation, by means of IL-1β secretion (3, 9) upregulate IL-1R in cardiac fibroblasts, in order to induce apoptosis, inflammation, and fibrosis. The NLRP3 inflammasome relates to an intracellular pattern recognition receptor that responds to pathogen-associated molecular patterns (PAMPs), or damage-associated molecular patterns (DAMPs), generating IL-1β and IL-18 and upregulating Smad 2/3 signaling, thus triggering cardiac inflammation, and fibrosis (6, 9, 28). In addition, it is likely that neutrophils obtain indirect pro-fibrotic properties by releasing extracellular traps (NETs) (3, 5, 6, 30). The NET release (NETosis) represents a response to different stimuli, whereas neutrophil elastase, and myeloperoxidase translocate from cytosolic granules into the nucleus, breaking down chromatin and the nuclear envelope, followed by a release of neutrophil content (30). Activated NETosis likely induces platelet accumulation, and activation, as well as secretion of pro-fibrotic mediators, such as TGF-β and PGDF, whereas fibroblasts exposed to NETs achieve the competence to proliferate, transdifferentiate, and produce collagens (3). Finally, neutrophils that undergo apoptosis generate anti-inflammatory signals (TGF-β, IL-10), aiming for a pro-resolving response (3, 29). Although neutrophils have a short life-span, evidence indicates that they persist after cardiac injury, taking part in ECM reorganization, thus enhancing cardiac fibrosis (3, 29, 30). These traits, if poorly regulated, make neutrophils potentially insidious, so supervision of neutrophil-initiated inflammation may be particularly important for the alleviation of the cardiac fibroproliferation feedback (3, 5, 29, 30).

Cardiac macrophages are cells essentially involved in signaling and cross-communication during homeostasis, or injury, *via* initiation, propagation, and the development of cardiac remodeling and fibrosis (1, 3, 5, 6, 31–33). Although substantial research has confirmed that the heart contains a wide phenotypic range of macrophages that mutually differ according to their origins and roles, the current immunometabolic field continues to widely recognize two main linages of macrophages, classified as M1 and M2 (3, 5, 6, 31–33). Initially, following a cardiac injury, monocyte-derived macrophages acquire an M1 phenotype, expressing the chemokine receptor CCR2, infiltrating injured cardiac tissue (5, 6, 26, 32, 33). These pro-inflammatory, phagocytic, and proteolytic macrophages secrete inflammatory cytokines (e.g., IL-1β, TNF-α, IL-6, monocyte chemoattractant protein (MCP)-1, NF-κβ, and ROS) (3, 5, 6, 32), due to their inflammatory gene expression, which includes genes implicated in the NLPR3 inflammasome generation (6). In contrast, the M2 phenotype, acquired in the subsequent (pro-regenerative/fibrotic) phase, releases anti-inflammatory cytokines (IL-10, lipoxins, and resolvins), and growth factors (TGF-β, vascular endothelial growth factor (VEGF), angiotensin II, fibroblast growth factor (FGF), and PDGF), to promote wound repair *via* myofibroblast activation, angiogenesis, and ECM protein deposition (6, 26, 31–33). The polarization of macrophages (M1 to M2) (3, 5, 6, 31, 32) is triggered by numerous stimuli such as hypoxia-inducible factor (HIF)-1α secretion, necrotic cardiomyocytes, damaged mitochondria, endothelial cell injury, MCP-1, neutrophil elastase, NETs, and ROS, and is likely obtained *via* the nuclear receptor subfamily 4 group A member 1 (NR4A1) (3). In addition to the monocyte-derived macrophages, a population of cardiac-resident macrophages is included in the reduced degree of cardiac damage (33). Their healing properties are likely induced by adopting different activation states and phenotypes during cardiac insult, presumably *via* the activation of mitogen-activated protein kinases (MAPKs), and MEK 1/2 (33), thus acquiring angiogenic, anti-inflammatory and anti-fibrotic properties (33).

Albeit experimentally induced cardiac fibrosis is highly associated with a significant increase in inflammatory macrophages (M1) evolution and accumulation, followed by decreased reparative M2-like polarization (6, 31, 32), both macrophages’ linages have the competence to convey pro- or anti-fibrotic traits (3, 5, 6, 33), thus exerting bidirectional regulation in cardiac inflammation and fibrosis (33). The M1 macrophages are rich in NLRP3 inflammasomes, and, when largely accumulated, produce generous amounts of IL-1β (9), thereby aggravating cardiac inflammation. The M2 phenotype likely undergoes macrophage to myofibroblast transition, *via* the TGF-β/Smad3 signaling pathway, to promote the generation of ECM proteins, thus further exacerbating fibrosis (33). Moreover, the M2 phenotype represents an abundant source of TGF-β, which therefore, *via* TGF-β-activated pro-fibrotic Smad, and non-Smad signaling, initiate cardiac fibroblast transdifferentiation (4, 7, 26, 33). Additionally, the associative data based on co-expression of different markers for identifying the origin of fibroblasts, implies that macrophages express the same non-specific protein as fibroblasts (fibroblast-specific protein-1), further indicating that they may be the relevant source of fibroblasts (1, 26). In terms of their anti-fibrotic characteristics, macrophages negatively regulate fibrosis by clearing apoptotic myofibroblasts, as well as cellular and matrix debris, and likely stimulate surrounding cells to produce MMPs, aiming for fibrosis resolution (26, 33). It can be argued that timely modulation of macrophage polarization may suspend some signaling between macrophages and fibroblasts, hence enabling appropriate and controlled fibroblast activation (26, 32, 33). Macrophages express remarkable functional plasticity, and their secretome possesses a powerful pro-fibrotic nature (1, 3, 5, 6, 31–33), whereas their particular “fibrogenic” macrophage lineage has not yet been identified (26), therapeutic manipulation of distinct macrophage phenotypes may prove to be relevant for appropriate repair functioning (31, 33). Accordingly, profound scrutiny is required for their targeted usage during cardiac injury.

Cardiomyocytes, upon injury, likely promote the setting for cardiac fibrosis, through the release of DAMPs, and, *via* neurohumoral and growth factor-mediated signaling network, activate inflammatory and pro-fibrogenic programming (1, 26). For instance, a bromodomain-containing protein 4 (BRD4) initiates a pro-fibrotic gene program in cardiomyocytes, for the expression of paracrine factors (e.g., plasminogen activator inhibitor-1 (PAI-1/Serpine1), and TGF-β2) that stimulates cardiac fibroblasts, and other stress-activated cell types, thus evoking a fibroproliferative response (8). Furthermore, cardiomyocytes themselves may acquire a myofibrogenic phenotype by secreting growth factors and matricellular proteins (1). Additionally, cardiomyocytes may favorably act upon fibroblast phenotype to restrain an otherwise fibrotic outcome (1).

From a pathophysiologic perspective, besides cellular effectors, several molecular pathways are involved in the initiation and progression of cardiac fibrosis: pro-inflammatory cytokines (TNF-α, IL-1β, and IL-6), chemokine cascades (MCP-1 and macrophage inflammatory protein (MIP)-2) (1, 26), and pro-fibrogenic growth factors (TGF-β and PDGF). However, TGF-β has been identified as the pivotal driver of fibroproliferation response in cardiac tissue, sustainably activated in cardiac fibrosis in humans, as well as in experimental models (1, 4, 7, 26). In a healthy heart, TGF-β is unable to associate with its receptors, however, after a cardiac insult, an almost immediate conversion from inactive to active forms occurs, and only a small quantity is sufficient to achieve the required cellular and signaling response (1, 26, 34, 35). Numerous mediators have been recognized as TGF-β activators, namely ROS, proinflammatory cytokines, and components of the extracellular matrix (26). The TGF-β function is multifaceted and context-dependent, acting as both a neutrophil and monocytes chemoattractant, whereas during the proliferative phase it acts as a crucial modulator of macrophage polarization toward the M2 subset, and as a suppressor of pro-inflammatory cytokines and chemokines (26, 35). Upon its up-regulation, TGF-β transduces signals *via* activation of intracellular cascades such as Smad-dependent (4), and non-Smad pathways (7, 34, 35). In the canonical TGF-β (classical) pathway, the activation of type II serine/threonine kinase receptors induces trans-phosphorylation of the type I receptor’s kinase domain, which in turn activates receptor-associated Smads, thus regulating the transcription of target genes (4). Multiple sources of data indicates that in cardiac fibroblasts, TGF-β, *via* Smads signals, induces fibroblasts transdifferentiation, α-SMA transcription, and subsequent ECM protein synthesis (e.g., collagens I, III, fibronectin), while simultaneously reducing protease-mediated matrix degradation (4, 26, 35). This particular pathway regulates the pool of cardiac fibroblasts through several subsequent processes, including the control of proliferation and apoptosis, modulation of fibroblast gene expression, initiation of cardiac myofibroblast activation by FoxO3a downregulation, and much more (4).

Beside the classical pathway, TGF-β functions in a Smad-independent manner through non-canonical mediators, including TGF-β-activated kinase 1 (TAK1), TNF-α, MAPKs, such as extracellular regulated protein kinases (ERK) 1/2, p38, and c-Jun amino terminal kinase (JNK), Rho kinases, phosphatidylinositol 3-kinase/protein kinase B (PI3K/Akt), NF-κβ, and some other signals (7, 34, 35). The non-canonical pathways are activated in order to boost, alleviate, supervise, and modulate TGF-β downstream cellular responses (7, 34, 35), operating in close partnership with Smad, as well as other non-Smad targets (34, 35). Each of these non-Smad signaling branches enables different cellular responses, including cell survival, protein synthesis, proliferation, and epithelial to mesenchymal transition (EMT) (34, 35). For instance, stimulation of p38 and JNK results in the phosphorylation of their transcription factors, with the intention to regulate differentiation, inflammation, and cell death (1, 7, 34). The p38, in particular, has been depicted as a key effector of MAPKs, and strong modulator of cardiac fibroblasts activity (34, 35). The p38α isoform, which is ubiquitously expressed in cardiac fibroblasts, positively regulates myocardial fibroblasts activation, whereas its deficiency results in an increased incidence of ruptured scar tissue and mortality following ischemic myocardial injury (7). These intracellular effectors (JNK, p38) may cross communicate with activated Smad components *via* their direct phosphorylation, thus aiming to regulate apoptosis and EMT. The up-regulation of ERK 1/2 is achieved through activation of Ras/Raf/MEK1/2 in a Smad-independent manner, whereby activated ERK 1/2, *via* phosphorylation of the target transcription factors, contributes to the EMT. TGF-β-induced PI3K/Akt up-regulation takes place due to an interaction between a p85 subunit of PI3K and the TGF-β receptor, and is significantly involved in TGF-β-induced fibroblast proliferation, morphological transformation, and the regulation of pivotal EMT transcription factors (34). Additionally, these non-Smad PI3k/Akt-mediated translational responses may specifically interact with Smad-mediated transcription, in order to protect cells from TGF-β-induced apoptosis in a context-dependent manner (34).

The process of epithelial to mesenchymal transition (EMT) represents one of the fundamental biological roles of TGF-β that is essential for embryonic development, but additionally represents a critical mechanism associated with fibrosis. Epithelial to mesenchymal transition refers to a complex cellular process (36–38), during which epithelial cells, stimulated by the signals gained from their microenvironment, acquire the phenotype and behavior of mesenchymal cells (myofibroblast). In chronic inflammatory settings, such as cardiac injury, the EMT process is initiated and mediated by a cascade of orchestrated signals that is activated by ROS, HIF-1α’ , TNF-α’ , and other proinflammatory cytokines, particularly TGF-β (34–38). The evidence confirms that TGF-β functions as a key molecular inducer of EMT molecular programming, through the activation of PI3K/Akt and Ras/MAPK pathways (6). Following the transition, these cells promote fibroblast-like morphology and architecture, with a tendency to migrate and invade surrounding tissue and the ability to produce ECM proteins (36–38). However, in the setting of persistent cardiac injury, extensive EMT contributes to the excessive formation of myofibroblasts, generating progressive cardiac fibrosis (38). Moreover, evidence indicates that the reversion of TGFβ-induced EMT in experimental models results in decreased fibrosis and appropriate healing (38). However, albeit successful *in vitro*, attempts at targeting EMT *in vivo* experimental models have so far failed, and continue to pose major challenges (38). Given that controlled TGF-β cardiac response is critical in order to maintain appropriate healing, distinct signaling cascades may be therapeutically targeted for the meticulous regulation of TGF-β programming. However, further research is needed to determine which pathways should be modulated, and if so, to what extent.

Considered as a whole, all the aforementioned pathways are directed toward the same purpose; fibroblast stimulation, hence their transdifferentiation into myofibroblasts (1, 3, 7, 26, 27, 39–41). Under physiological conditions, cardiac fibroblasts are quiescent structures; thus, in the setting of diverse heart injuries, these cells acquire an entirely new state (1, 3, 7, 26, 27, 39–41), expressing a strong pro-inflammatory, hyper-secretory, and hyper-migratory phenotype (7). Accordingly, myofibroblasts express contractile proteins, such as α-smooth muscle actin (α-SMA, and the ACTA2 gene) (1, 4, 27, 39), fibrillar collagens, and matricellular proteins (1, 4, 7, 26, 27, 39–41). Besides fibroblasts dwelling in the healthy interstitium of the heart (∼10–20% of the cardiac cell population) (39, 40), which are highly prone to up-regulation, macrophages, cardiomyocytes, epithelial, and endothelial cells likely convert to fibroblasts or myofibroblasts, thus boosting the myofibroblast pool in injured hearts (1, 26, 39, 40). Upon transdifferentiation, myofibroblasts express several specific traits, such as multiple dendritic processes, elongated and serrated nuclei, extensive rough endoplasmic reticulum, and the formation of an extensive stress-fiber network (39). In addition, these cells synthetize and secrete ECM proteins such as collagens (e.g., I, III), a specialized isoform (splice-variant) of fibronectin, periostin, and actively express cadherin-2 and -11 (39). Myofibroblasts also undergo metabolic remodeling, with regard to the energetic needs of a proliferating/differentiating cell. Therefore, an increase in aerobic glycolysis causes oxidative metabolism to occur in order to support the newly obtained phenotype (39). For instance, increased glycolysis and the decreased oxidation of glucose boosts the production of lactate, which induces myofibroblast marker expression (α-SMA, fibronectin, and collagens). Moreover, lactate represents an initiation signal for macrophage polarization toward M2-like phenotypes, as well as further production of profibrotic cytokines (39). Finally, scar formation is followed by myofibroblasts’ new morphology, identified as matrifibrocyte, expressing genes associated with bone and cartilage remodeling (Cilp2 and Comp) (40). Myofibroblasts likely undergo apoptosis, or senescence, or may revert to their initial state of sedentary fibroblasts (40), whereas the recognition of fibroblasts competence, to “revert” to the quiescent phenotype, gives rise to the possibility of reverse remodeling for cardiac fibrosis (7).

The comprehensive crosslink of various-domains cells, as well as the vast signaling network that interconnects them, results in the scarcity of effective anti-fibrotic treatments, while current clinical trials disclose unsatisfactory anti-fibrotic therapeutic results (41). Only the most meticulous analysis of all the processes involved in the cardiac fibrosis pathophysiology may aid us in the identification, and recognition of the most feasible targets, that may become beneficial for therapeutical modulation.

## Renalase’s Structure, Expression, and Cell Biology

Renalase is identified to be a flavin adenine dinucleotide (FAD)-dependent amine oxidase, introduced into the scientific world in 2005 (42). Its discovery is based on the hypothesis that kidneys secrete some additional proteins exerting relevant biological roles, particularly with the intention of maintaining renal and cardiovascular health (42, 43). Moreover, the observation that patients with chronic kidney disease experience an increased risk of developing cardiovascular complications, including cardiac hypertrophy and fibrosis, conditions that are highly associated with sustained inflammation and oxidative stress, poses the important question of whether there is a molecule capable of mitigating these deleterious events and their related consequences. Indeed, after a comprehensive analysis of 114 candidate genes encoding novel secretory proteins (42), one clone was determined to be preferentially expressed in the proximal renal tubules and cardiomyocytes, as well as in the liver and skeletal muscles (42). According to its significant kidney distribution and presumed enzymatic activity, the newly identified protein was named renalase (42). Additional studies provided evidence that renalase’s distribution is spread far beyond the kidneys and heart. Therefore, its tissue expression pattern, to varying extents, is revealed in the liver (19, 44), small intestines (45), skeletal muscles (46), pancreas (22, 47), cornea (48), brain and peripheral nerves (49, 50), and malignant tissue as well (22, 23, 51), implying renalase’s remote role in general homeostasis and its possible relevant biologic activity. Accordingly, the latest research indicates a potential role for renalase in placental development and function, owing to its expression in the placenta throughout human gestation (25).

The concept of renalase’s pathophysiological relevance spurred the identification of its biochemical and pathophysiological nature, providing the following conclusions. The human renalase gene is located on chromosome 10, and it contains 11 exons (10, 42, 43). There are two major isoforms of renalase in humans, known as renalase 1, and 2 (52), and two more isoforms (3 and 4), that lack oxidase function activity due to their significantly shortened amine oxidase domains (10, 13, 43). It has to be noted that the existence of these variants significantly impedes the identification of renalase-dependent regulations and mechanisms of action, which to date remain not fully understood. The most recent evidence, however, clearly shows that renalase’s key structural features include a putative signal peptide at the N terminus, which is likely cleaved in secreted forms, a FAD-binding domain, and a nicotinamide adenine dinucleotid phosphate NAD(P)H oxidase domain (10, 42, 43). Renalase protein is similar, but not identical to the monoamine oxidase (MAO)-A and MAO-B, sharing only 14% of their amino acid identity (52). However, it is revealed that it metabolizes catecholamines and catecholamine-like substances (42, 43, 53), *via* a superoxide (O2^–^)-dependent mechanism, with nicotinamide adenine dinucleotide phosphate (NAD(P)H) as a co-factor. More recent research confirms that renalase converts endogenous dihydro forms of β-NAD(P)H to metabolically available, non-inhibitory β-NAD(P)H form (54, 55). Moreover, up until now, renalase seems to be the only identified amine oxidase that is secreted into the blood, circulating at a concentration of approximately 3–5 μg/ml, and by metabolizing the circulating catecholamines, exerts significant hypotensive effects (11, 43, 53). Indeed, renalase has been proven to be implicated in numerous cardiovascular conditions, including chronic heart failure, coronary artery disease, hypertension, diabetes mellitus, aortic stenosis (56–63), and renal pathologies (64–66), suggesting potential benefits in the course of the cardio-renal axis.

Such results set the stage for renalase’s pathophysiology to be moved to the next round of research, far beyond its catalytic activities. Indeed, in recent years, renalase has been established as a molecule exerting anti-inflammatory and antiapoptotic actions, with the intention of cell survival, and that significantly protects organs and tissues, independently of its enzymatic traits (10–25, 44–48). Surprisingly, tumor cells use renalase’s action as a survival strategy (10, 11, 51, 67). Evidence indicates that renalase’s tissue expression can be regulated, presumably as a response to local and systemic environmental factors, for protection. Moreover, as shown, renalase’s gene expression is regulated by several transcription factors, such as HIF-α’ 1, Sp1, STAT3, ZBP89, TNF-α’ , NF-κβ (10, 11), several of which are associated with proinflammatory responses.

Once up-regulated, renalase (and renalase-derived peptides) provide their pleiotropic biologic activities *via* the receptor identified to be the plasma membrane Ca^2+^-ATPase 4b (PMCA4b) (10, 11, 16, 25). The PMCA4b is a broadly shared low-capacity plasma membrane calcium transporter that seems to mediate the effects of renalase in diverse tissues. The factor of note is that signals elicited by renalase trigger a prosurvival signaling circuit, with the activation, or inhibition, of phosphatidylinositol 3-kinase/protein kinase B (PI3K/Akt), extracellular signal-regulated kinase (ERK), p38 mitogen-activated kinase, B cell lymphoma 2, and inhibition of c-Jun N-terminal kinase (JNK) (10, 11, 13). Moreover, renalase also evokes important downstream pathways *via* the same receptor, namely Ras/Raf/MEK/ERK, p38, NOS, NF-κβ, cAMP, and PI3K/Akt pathways (10). The aforementioned findings implicate renalase as a significant molecule for targeting diverse signaling pathways related to inflammation, oxidative stress, apoptosis, and fibrosis. Accordingly, the proposed molecular signature of renalase captured the interest of current investigators in defining the precise mechanism of renalase’s action and, if proved relevant, translating it into a clinical setting. Therefore, it will be critical to conduct research toward a more detailed understanding of renalase’s pathophysiology.

## The Identification of Renalase as a Relevant Antifibrotic Molecule Within the Context of Cardiac Fibrosis

**FIGURE 1:**
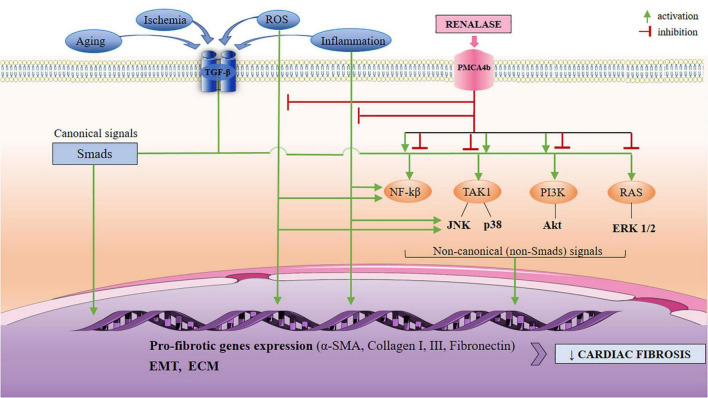
A schematic view of the TGF-β-mediated activation of non-Smad (non-canonical) pathways, with respect to anticipated signaling arms that may be targeted by renalase. The non-canonical network which includes the MAPKs (ERK 1/2, JNK, p38), PI3K/Akt, and NF-κβ may all be modulated by renalase, at different levels: renalase inhibits or activates phosphorylation and the activation of p38, and PI3K/Akt pathways; it also downregulates phosphorylation of ERK 1/2, and JNK, and is up-regulated by NF-κβ. The mediation of the TGF-β non-canonical signaling by renalase is likely context-dependent to regulating EMT, and pro-fibrotic gene expression (α’ -SMA, collagens I, III, fibronectin), and results in increased ECM, and diminished MMP-1 expression. Based upon current data, renalase does not mediate the canonical (Smad2/3) network. TGF-β, transforming growth factor-β; PMCA4b, plasma membrane calcium-ATPase 4b; MAPKs, mitogen-activated kinases; ERK 1/2, extracellular regulated protein kinases 1/2; JNK, c-Jun N-terminal kinases; PI3K/Akt, phosphatidylinositol 3-kinase/protein kinase B; NF-κβ, nuclear factor κβ; EMT, epithelial to mesenchymal transition; α-SMA, α-smooth muscle actin; ECM, extracellular matrix.

In light of the pivotal underlying mechanisms of myocardial fibrosis validated by the fibroblast-specific, *in vivo* model (7), the particular focus herein is on the TGF-β and mitogen-activated kinases (MAPKs) pathways, which are evidenced targets by renalase (11, 13, 16, 17, 21, 68, 69), as presented in Figure 1.

Any type of injury, including myocardial, challenges a complex intracellular signaling cascade, with precise intersection points, to elicit repair mechanisms, and is mostly achieved through the activation of TGF-β (1, 4, 7, 26, 35, 70–74). Although TGF-β may be secreted by various cell types (macrophages, fibroblasts, cardiomyocytes, and platelets), its signaling in the injured heart is predominantly accomplished *via* its activation (1, 26, 35, 71–73). TGF-β pro-fibrotic properties are primarily achieved by inducing fibroblast transdifferentiation into highly contractile myofibroblasts (1, 4, 7, 26, 35, 72–74); thus, neo-expression of the α-SMA isoform (72) regulates macrophage function (72–74), promotes EMT (37, 38, 75, 76), and inhibits epithelial cell growth and migration (72). Among the pro-fibrotic TGF-β-driven molecular signals, as mentioned previously, MAPKs signaling is among the best-characterized positive regulators of fibroblast activation and myocardial fibrosis (7, 34, 35, 77, 78). It consists of classical cascades as follows: extracellular signal-regulated kinases (ERK) 1/2, c-Jun N-terminal kinases (JNK), and p38 (7, 26, 34, 35, 77, 78), whereas the mounting body of research indicates the detrimental role of p38 and ERK 1/2 (7, 79–83) in cardiac fibrosis pathogenesis. Some evidence suggests that p38 inhibition is followed by a reduction in TGF-β-induced expressions of collagen I and α-SMA in cardiac fibroblast culture (79). Genetic manipulation of the p38 *in vivo* demonstrates reduced α-SMA expression, suggesting that p38 lies upstream of the α-SMA expression in fibroblasts, indicating this pathway as a key regulator of cardiac fibrosis (81). The pharmacological inhibition of p38 in the culture of rat cardiac fibroblasts abolishes α-SMA expression, further verifying p38α as the essential player for α-SMA expression in heart fibroblasts (82). On the other hand, the interaction between ERK 1/2 and p38 signals has also been acknowledged to significantly promote cardiac fibrosis (82). Accordingly, TGF-β-induced cardiac fibrosis is suggested to be initially mediated by ERK 1/2 phosphorylation *via* Rho-kinase 1 activation (83), or induced IL-6 expression (80), and that fibrosis may be effectively alleviated by the hindering of the ERK 1/2 pathway. Taken together, the strategies that specifically target cardiac TGF-β-mediated MAPKs (Smad-independent) signaling (7, 77–79) offer some benefit in lowering the risks of fibroproliferative response.

Several lines of evidence suggest that recombinant renalase substantially mediates non-canonical TGF-β (Smad-independent) pro-fibrotic pathways, in experimentally induced heart and kidney fibrosis (68, 69, 84), including diabetic nephropathy (17), and pressure overload-induced heart failure (21), presuming that if treated with renalase, it results in an appropriate, therefore plausible fibrotic feed-back. As clearly demonstrated, systemic renalase administration significantly alleviates experimentally induced fibrosis of both, the heart and kidneys, and prevents adverse tissue remodeling. Moreover, a few pertinent pathways of renalase protection are offered that may be clinically relevant. The research (84) demonstrates that in rats subjected to subtotal nephrectomy, renalase supplementation exerts several favorable responses. In the heart, in particular, renalase administration mitigates cardiomyocyte hypertrophy, decreases systolic blood pressure, decreases left ventricular (LV) end-diastolic posterior wall thickness, and LV end-diastolic pressure, and restores cardiac diastolic function. More importantly, renalase treatment significantly alleviates cardiac interstitial fibrosis, followed by the increased cardiac expression of MMP-1, lower expression of TIMP-1, and TGF-β, and decreased phosphorylated ERK 1/2 levels. Similar effects of renalase administration are obtained in the remnant kidney, also evidenced as the amelioration of kidney function, lessened degree of glomerular sclerosis and tubulointerstitial fibrosis, and significantly downregulated expression of fibrosis markers (collagen I, collagen III), TGF-β, pro-inflammatory cytokines (IL-6, TNF-α’ , and MCP-1), NADPH oxidative stress components (gp91^phox^, p47^phox^, p67^phox^) and suppression of phosphorylation of ERK 1/2, corresponding to that in the heart (84). Moreover, the administration of renalase in rats subjected to 5/6 nephrectomy (24) results in a significant decrease in arterial pressure, LV hydroxyproline concentration, as a measure of fibrosis, and noradrenaline levels, and in amelioration of LV/body weight ratio, and LV papillary muscle tension. In a similar manner, in rats with diabetic nephrosclerosis, renalase supplementation significantly attenuates high glucose-induced profibrotic gene expression, and p21 expression *via* the impediment of ERK 1/2 signals (17). A more recent study reveals that recombinant renalase significantly mitigates pressure overload-induced cardiac failure, being highly associated with p38 and ERK 1/2 signaling (21). With reference to the aforesaid data, related research of renal fibrosis (69) indicates that renalase treatment downregulates the expression of α’ -SMA, collagen I, and fibronectin, thereby hindering renal interstitial fibrosis. The potential for renalase to downregulate α’ -SMA, a contractile protein expressed by activated myofibroblasts, fibronectin (provisional matrix protein), and collagen I (major fibrillar collagen), while restoring the expression of E-kadherin (epithelial adhesion receptor), implies that renalase administration may prevent, or even suppress interstitial fibrosis in tubular epithelial cells (69), offering the scientific rationale that the same effects may be obtained in the regulation of cardiac α’ -SMA transcription, and prevention of myofibroblast conversion. Moreover, the research which suggests that renalase significantly suppresses the expression of phospho-ERK 1/2 in TGF-β stimulated cells, and that *via* suppression of the ERK 1/2 signals impedes the process of TGF-β-induced EMT essentially corresponds to the proposed pathogenesis of cardiac fibrosis, thus deserving a more detailed consideration. Nevertheless, as stated, the EMT refers to a phenomenon that is, based on an increasingly developed body of evidence, recognized to be similarly involved in the pathogenesis of cardiac fibrosis (36–38, 85–88). Numerous soluble factors, predominantly TGF-β (36–38, 86–88), and extracellular matrix components act synergistically to elicit EMT programming in various tissues, including the heart, whereas MAPKs signals (p38, ERK 1/2, and JNK), upregulate transcription factors for epithelial-mesenchymal transition (87, 88). Additional evidence that renalase *(in vivo)* inhibits EMT *via* direct suppression of ERK 1/2 signaling (17, 21, 68, 69, 84), likewise in the aforementioned research, indirectly impeding the source for myofibroblasts, suggests scientific plausibility for renalase to be involved in cardiac fibrosis scrutiny. In addition, the downregulation of renalase signals is verified to be associated with sustained activation of p38, and *vice versa* (51), further implying the inverse association of renalase and MAPKs signaling network. In line with that, the partial inhibition of MAPKs, after a cardiac insult, promotes cardiomyocytes and endothelial cells proliferation, resulting in reduced scarring and cardiac fibrosis (89–91), whereas the deleterious role of MAPKs pathway in ECM remodeling is promoted *via* TNF-α, and IL-6 upregulation in cardiomyocytes, and the induction of TGF-β signaling, thus fibroblast transdifferentiation (91, 92). However, the upregulation of MAPKs in injured or remodeling myocardium, regardless of the pathophysiological stimuli, results in fibroblast activation and profibrotic feedback (1, 7, 77, 78, 91, 92). Albeit the vast majority of evidence underscores the deleterious effects of p38 activation, their pharmacological inhibition, although theoretically very attractive, did not support any efficacy in clinical trials (93, 94).

Nevertheless, the precise mechanism of renalase’s anti-fibrotic effects, and its crosstalk with the MAPKs pathways is just beginning to emerge, though, to date, few concerns need further clarification. For instance, some research evidences that renalase alleviates fibrosis by inhibiting the activation of MAPKs (17, 21, 69, 84), whereas other findings indicate that renalase exerts cytoprotection *via* activation of MAPKs transduction pathways: PI3K/Akt, ERK 1/2, and p38 signaling (13, 16). Such conflicting results may be explained by the possible “duality” of renalase’s actions, presenting with context-dependent signaling (i.e., state of the target cell, experimental conditions, time-dependent, or different tissue), and by its ability for context-related adjustments. The unique conclusion of all preclinical studies, *in vitro*, and *in vivo*, however, is that renalase, after binding to its receptor (PMCA4b), sets in motion several molecular pathways which aim to modulate “renalase-dependent” MAPKs signaling, in order to exert pleiotropic, presumably context-dependent actions toward cytoprotection.

Respectively, in the context of renalase’s diverse roles, renalase’s additional renal anti-fibrotic mechanisms of action are identified (68), offering expertise that may be relevantly expanded to the pathophysiology of cardiac fibrosis. Renalase administration, however, abolishes malondialdehyde (MDA), and promotes superoxide dismutase (SOD) expression, whereas, in addition, it diminishes the oxidative stress-induced expression of α’ -SMA, fibronectin and collagen-I, and restores E-cadherin expression, in a dose-dependent manner (68). These results clearly implicate that renalase, if administered in a timely manner, mitigates oxidative stress, thus aiming to postpone the deleterious effects of ROS, particularly the onset of the EMT that, as noted, contributes to fibrosis. Nevertheless, *in vitro* studies show that increased oxidative stress plays a critical role in TGF-β-induced EMT *via* MAPKs activation, resulting in renal interstitial fibrosis (70, 71, 95). Similarly, the pharmacological inhibition of ROS generation, and inactivation of MAPKs pathways alleviate TGF-β-induced EMT in bronchial epithelial cells, resulting in minimized pulmonary fibrosis (71, 96). If it is acknowledged that renalase inhibits the phosphorylation of ERK 1/2, as evidenced (17, 21, 69, 84), whose activation, in particular, results in increased oxidative stress, it is then reasonable to believe that renalase, by suppression of ROS generation, indirectly inhibits, at least to some extent, the process of EMT, and protects against fibrosis. A theoretically similar approach, by direct targeting of the molecular mechanisms that underlie EMT, has been suggested as a novel opportunity for an effective anti-fibrotic therapy (38, 85, 97). Finally, the related research of renalase’s effects on fibrosis markers in the liver-injury mouse model (19) further confirms that the lack of the renalase gene favors oxidative stress promotion, significant macrophage accumulation, as discussed, and increased TGF-β expression, finally emerging as liver fibrosis progression. A schematic view of the TGF-β-mediated activation of non-canonical pathways with respect to signaling arms that may be targeted by renalase is depicted in Figure 1, and a summary of current experimental evidence is presented in Table 1.

**TABLE 1:** A summary of the effects of renalase based upon current experimental evidence.

Outcome	Experimental model	Major findings	Therapeutic context	References
Anti-necrosis Anti-apoptosis Anti-oxidation Anti-inflammation	Contrast-induced nephropathy	Renalase pretreatment provides protection against CIN *via* renal function amelioration, while suppressing tubular necrosis, apoptosis, oxidative stress, and inflammation.	Reno-protection in patients receiving contrast	(12)
Anti-necrosis Anti-apoptosis Anti-inflammation	Cisplatin-induced AKI	A deficiency of renalase leads to significant renal tubular necrosis, apoptosis, and macrophage infiltration *in vivo*, while renalase treatment *in vitro* reduces necrosis, and apoptosis *via* PI3K/Akt, and MAPK activation, as well as JNK downregulation.	AKI protection in patients receiving cisplatin therapy	(13)
Anti-oxidation	Cisplatin-induced AKI	Renalase administration *in vivo* and *in vitro* regulates ROS generation, mitochondrial dynamics, and SIRT3 levels, providing protection in cisplatin-induced AKI.	Reno-protection in patients receiving cisplatin therapy	(14)
Anti-hypertension Anti-hypertrophy Anti-inflammation	Diabetic nephropathy	The downregulation of renalase results in hypertension, albuminuria, mesangial hypertrophy, renal inflammation, and injury, whereas renalase administration mitigates high glucose-induced profibrotic gene expression, and p21 expression *via* the inhibition of ERK 1/2 signaling.	Mitigation of diabetic nephropathy progression	(17)
Anti-necrosis Anti-apoptosis	MIRI	Renalase pretreatment significantly reduces myocardial cell necrosis and apoptosis in myocardial ischemia reperfusion injury.	Therapy for cardiac ischemia/IR injury	(18)
Anti-oxidation Anti-inflammation Anti-fibrosis	Non-alcoholic steatohepatitis	Oxidative stress, macrophage infiltration, Adgre1 (a marker of mature macrophages), and TGF-β1 expression are significantly increased in the absence of renalase in non-alcoholic steatohepatitis *in vivo*.	Mitigation of liver fibrosis progression	(19)
Anti-necrosis Anti-inflammation Anti-fibrosis	Cisplatin-induced chronic kidney disease	Kidney-targeted renalase agonist supplementation ameliorates levels of plasma creatinine, decreases inflammatory cytokines levels, inhibits kidney necrosis, restores nephron epithelia and vasculature, while acting to suppress inflammatory macrophages and myofibroblasts.	Reno-protection in patients receiving cisplatin therapy	(20)
Anti-hypertrophy	Pressure overload-induced HF	Recombinant renalase significantly alleviates the pressure overload-induced cardiac failure *via* p38 and ERK 1/2 signaling pathways.	Mitigation of heart failure progression	(21)
Anti-hypertension Anti-hypertrophy Anti-fibrosis	Chronic kidney disease	Renalase supplementation decreases mean arterial pressure, LV/body weight ratio, LV hydroxyproline concentration (a measure of fibrosis), and noradrenaline levels, resulting in the significant decrease of LV papillary muscle-developed tension.	Amelioration of cardiac function and blood pressure in CKD	(24)
Anti-apoptosis Anti-oxidation Anti-inflammation	Contrast-induced nephropathy	Limb IPC-induced reno-protection in CIN results in amelioration of the renal function and tubular damage, reduction of renal oxidative stress and inflammation, and significantly relies upon renalase up-regulation *via* activation of the TNF-α/NF-κβ pathway.	Reno-protection in patients receiving contrast	(32)
Anti-oxidation	Hepatic IR injury	Renalase is highly sensitive and responsive to oxidative stress, both *in vitro* and *in vivo*, which may be suppressed by antioxidant treatment.	Biomarker and protection in hepatic IR Injury	(44)
Anti-oxidation	Small intestine and Caco-2 oxidative injury	Small intestinal renalase expression is mediated by NF-κβ p65 and is upregulated in fasting-induced oxidative stress.	Anti-oxidative intestinal protection	(45)
Anti-inflammation	Cerulein-induced acute pancreatitis	A genetic deficiency of renalase increases the development of pancreatitis, whereas renalase treatment reduces pancreatic injury, as well as neutrophil and macrophage infiltration, through activation of its receptor, PMCA4b.	Acute pancreatitis therapy	(47)
Anti-oxidation Anti-fibrosis	Unilateral ureteral obstruction	Renalase administration blocks oxidative stress-mediated EMT, by effectively decreasing α-SMA expression, fibronectin and collagen I, while restoring the expression of E-cadherin and interstitial fibrosis.	Mitigation of CKD progression	(68)
Anti-fibrosis	Unilateral ureteral obstruction	Renalase administration *in vivo* maintains E-cadherin expression and suppresses the expression of α-SMA, fibronectin and collagen I. Renalase administration *in vitro* has been shown to inhibit TGF-β-mediated upregulation of α-SMA and downregulation of E-cadherin, and ameliorate renal interstitial fibrosis by impeding tubular EMT *via* the suppression of the ERK 1/2 signaling network.	Mitigation of CKD progression	(69)
Anti-hypertension Anti-hypertrophy Anti-oxidation Anti-inflammation Anti-fibrosis	Subtotal nephrectomy	Renalase supplementation reduces hypertension, cardiomyocytes hypertrophy, and cardiac interstitial fibrosis, proteinuria, glomerular hypertrophy, and interstitial fibrosis. Renalase decreases the expression of proinflammatory cytokines (TNF-α, and IL-6), macrophage infiltration, activation and polarization, NADPH oxidase components (gp91^phox^, p47^phox^, and p67^phox^), the expression of collagen I, III, TIMP-1, and TGF-β1, and increases the expression of MMP-1. It also prevents cardiac remodeling through the inhibition of ERK 1/2 phosphorylation and suppresses pro-fibrotic gene expression.	Cardiovascular and renal protection in patients with CKD	(84)
Anti-hypertension	Response of HK-2 cells to epinephrine	Epinephrine acts to stimulate renalase secretion through α-adrenoceptor/NF-κβ pathways within renal proximal tubular epithelial cells.	Hypertension treatment in CKD	(101)
Anti-oxidation	Treadmill exercise	Renalase expression is regulated by NF-κβ in the plantaris muscle, and renalase expression is increased as the result of acute exercise-induced oxidative stress.	Anti-oxidative muscle protection in acute exercise	(105)
Anti-necrosis Anti-apoptosis Anti-inflammation	Ischemic AKI	Renalase treatment attenuates the increase in creatinine plasma levels, reduces catecholamine levels, abolishes renal tubular necrosis, apoptosis, neutrophils and macrophage infiltration. A deficiency of renalase increases the expression of proinflammatory genes (TNF-α, MCP-1, and MIP-2).	Biomarker, prevention and therapy for AKI	(106)
Anti-necrosis Anti-apoptosis Anti-oxidation Anti-inflammation	Fatty liver IR injury	Renalase administration alleviates necrosis and apoptosis of liver tissue, decreases ALT, AST, and LDH plasma levels, suppresses ROS generation, and effectively mitigates mitochondrial damage in fatty liver IR injury *via* SIRT1 upregulation.	Attenuation of liver IR injury	(117)
Anti-ischemia Anti-necrosis	Renalase-knockout mice and isolated perfused hearts	Renalase deficiency, *in vivo*, results in left ventricular hypertrophy, elevated blood pressure, electrolyte disturbances, and a significant degree of myocardial necrosis, whereas renalase application *in vitro* provides cardio protection by functioning as plasma NADH oxidase, effectively regulating extracellular NAD^+^ levels.	Cardio-protection in CKD	(118)

*CIN, contrast-induced nephropathy; AKI, acute kidney injury; PI3K/Akt, phosphatidylinositol 3-kinase/protein kinase B; MAPK, mitogen-activated protein kinase; JNK, c-Jun N-terminal kinase; ROS, reactive oxygen species: SIRT, sirtuin; ERK1/2, extracellular regulated protein kinases 1/2; MIRI, myocardial ischemia reperfusion injury; IR, ischemia/reperfusion; Adgre1, adhesion G protein-coupled receptor E1; TGF-β, transforming growth factor-β; HF: heart failure; LV: left ventricular; CKD, chronic kidney disease; IPC, ischemic preconditioning; NF-κβ, nuclear factor κβ; Caco-2, human colorectal adenocarcinoma cells; PMCA4b, plasma membrane calcium ATPase 4b; EMT, epithelial-mesenchymal transition; α-SMA, α-smooth muscle actin; NADPH, nicotinamide adenine dinucleotide phosphate; TIMP-1, tissue inhibitor of metalloproteinase-1; MMP-1, matrix metalloproteinase-1; HK-2 cells, human proximal renal tubular epithelial cells; MCP-1, monocyte chemoattractant protein-1; MIP-2, macrophage Inflammatory protein 2; ALT, alanine transaminase; AST, aspartate aminotransferase; LDH, lactate dehydrogenase; NAD, nicotinamide adenine dinucleotide.*

Notwithstanding the limited experimental information, it may be possible that the supervising of several pro-fibrotic steps by one multifaceted molecule, such as renalase, may be an initial or adjunct approach toward fibroproliferative drive regulation. These promising results call for larger preclinical research of renalase regarding its potential benefits for the prevention of cardiac fibrosis in diseased hearts.

## The Pathophysiological Relevance of Renalase as an Anti-Inflammatory, Anti-Apoptotic and Anti-Oxidative Molecule in the Context of Cardiac Fibrosis

**FIGURE 2:**
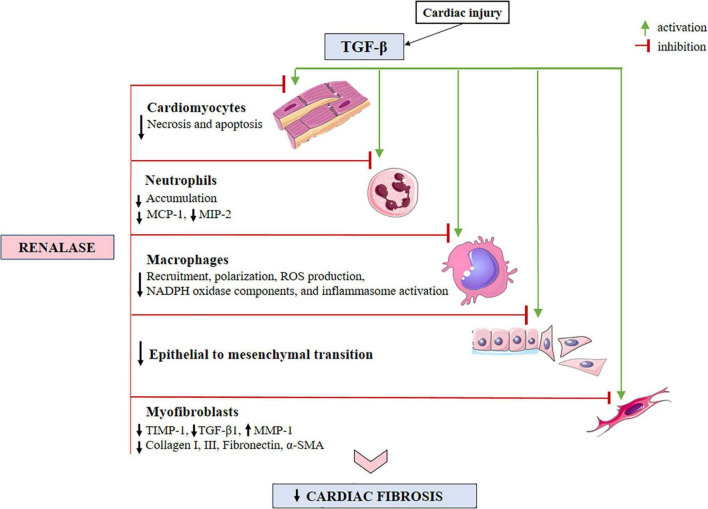
A schematic view of the TGF-β-activated cellular effectors, with respect to anticipated sites for renalase action within the injured myocardium. Evidence has shown that renalase both inhibits necrosis and apoptosis, while diminishing neutrophil infiltration. Renalase lessens total macrophage accumulation, particularly the M1-like (pro-inflammatory) sub-type, and likely serves to promote M2-like (anti-inflammatory) phenotypes, suppressing inflammasome activation. Additionally, renalase downregulates the expressions of MCP-1, MIP-2, and NADPH oxidase components (gp91^phox^, p47^phox^, and p67^phox^), suppresses epithelial to mesenchymal transition, and decreases the expression of α-SMA, collagen I, and III, TIMP-1 and TGF-β-1, while increasing the expression of MMP-1. TGF-β, transforming growth factor-β; MCP-1, monocyte chemoattractant protein*-*1; MIP-2, macrophage inflammatory protein-2; ROS, reactive oxygen species; NADPH, nicotinamide adenine dinucleotide phosphate; TIMP-1, tissue inhibitor of metalloproteinase-1; MMP-1, matrix metalloproteinase-1; α-SMA, α-smooth muscle actin.

**FIGURE 3:**
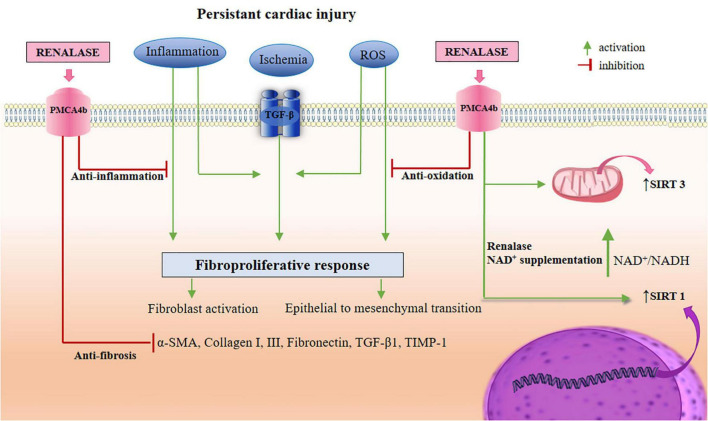
A schematic view of renalase-targeted pathways in the TGF-β-induced cardiac fibrosis. As depicted in the Figures 1, 2, renalase likely regulates the actions of signaling branches in non-canonical pathways (ERK 1/2, JNK, p38, and PI3K/Akt), and several cellular effectors, in order to establish a state of anti-necrosis, anti-apoptosis, anti-inflammation, anti-oxidation, suppression of EMT, and anti-fibrosis. Simultaneously, renalase promotes the activation of SIRT1 *via* NAD^+^ supplementation, which therefore presumably provides an indirect role in SIRT1 cardio-protection. These actions include: the reduction of ROS production, inflammation, cardiomyocytes apoptosis, and cardiac fibroblasts transdifferentiation, as well as increased autophagy, and fatty acid oxidation, thus inducing mitochondrial biogenesis. In addition, renalase, with NAD^+^ supplementation, promotes SIRT3 activity, thus potentially upgrading SIRT3 cardio-protective properties, including: the alleviation of cardiomyocytes apoptosis, hypertrophy, and fibrosis, suppression of ROS production, and inflammation, extinguishing MAPK/ERK 1/2, and PI3K/Akt signaling, and EMT, which thereby increases autophagy for inflammasome suppression, and promoting mitochondrial energy production. TGF-β, transforming growth factor-β; PMCA4b, plasma membrane calcium-ATPase 4b; ERK 1/2, extracellular regulated protein kinases 1/2; JNK, c-Jun N-terminal kinases; PI3K/Akt, phosphatidylinositol 3-kinase/protein kinase B; EMT, epithelial to mesenchymal transition; SIRT, sirtuin; NAD, nicotinamide adenine dinucleotide; ROS, reactive oxygen species; MAPK, mitogen-activated protein kinase.

It is known that any kind of tissue damage involves mechanisms such as inflammation, oxidative stress, apoptosis, metabolic disturbances, and fibrosis. Pathophysiologically speaking, if anti-fibrotic therapy aims at its highest efficiency, it should evenly and meticulously address inflammation, while simultaneously providing a wide array of anti-fibrotic actions. Significant progress in understanding renalase’s pathophysiology over the last few years has been achieved owing to the data obtained from cell cultures and organ-damaged animal models, warranting that renalase (*in vitro* and *in vivo)* encompasses the pivotal pathways of cell trauma: inflammation, oxidative stress, necrosis, and apoptosis. These findings have helped to establish several hypotheses and concepts that renalase, by its successive or simultaneous acting on various cells, and eliciting different signals, may be essentially involved in the pathogenesis of cardiac fibrosis (Figures 2, 3). Such a knowledge base, perhaps, offers some feasibility that renalase supplementation, during the course of various cardiac injuries, presumably provides some beneficial anti-fibrotic effects. Accordingly, in the following sub-section, we will specifically focus on the effects of renalase that have been validated *in vitro* and *in vivo* and may be implemented in the pathogenesis of cardiac fibrosis.

Regarding the context of inflammation and the proposed anti-inflammatory roles of renalase, it should be noted that renalase gene expression is upregulated by transcriptional factors, several of which have been linked to proinflammatory actions, most notably NF-κβ and hypoxia-inducible factor (HIF)-1α (10, 98–102). As a result, the NF-κβ has been scrutinized as the master regulator of inflammation and immune homeostasis, with a variety of relevant pro- and anti-inflammatory factors under its transcriptional control (103, 104), renalase clearly being one of them (45, 100–102). Furthermore, in addition to being a powerful inflammatory response driver, NF-κβ acts as a regulator of anti-apoptotic gene expression, and oxidative mechanisms modulation, and it can be activated by pro-inflammatory signals, and hypoxia (HIF-1α) to inhibit apoptosis and promote cell survival (104). Based on the evidence indicating renalase as a prosurvival and anti-inflammatory protein (10–13, 20, 45, 47, 84, 101, 102), it is possible that these protective effects are obtained *via* TNF-α/NF-κβ axis-mediated up-regulation of renalase expression (10, 11, 45, 101, 102), whereas pharmacological inhibition of TNF-α (by Humira), and NF-κβ (by pyrrolidine dithiocarbamate) signaling accordingly downregulates renalase expression, leaving the tissues partially unprotected (102). Furthermore, renalase systemic administration reduces TNF-α concentration (12, 84, 105, 106), which may be clinically relevant in the context of cardiac fibrosis, given that a plethora of *in vivo* evidence suggests that TNF-α plays a significant role in the pathophysiology of cardiac fibrosis. TNF-α, for example, has been shown to correlate positively with collagen turnover markers (26), to initiate the synthesis of various ECM proteins followed by fibroblast proliferation (107), to induce fibrogenic activation of immune cells toward a fibrotic phenotype (2, 3, 5–7), and to induce TGF-β expression (108), for further fibroblast transdifferentiation.

The evidence that epinephrine significantly increases renalase secretion in renal proximal tubular epithelial cells cultured *via* α-adrenoreceptor/NF-κβ pathways (101), and in the intestinal cells (45) provides another theoretical explanation for its anti-inflammatory actions. For instance, the secretion of pro-inflammatory IL-6 is, in a similar manner, obtained by the stimulation of the same α-adrenergic receptors stimulation through p38 MAPK, and the NF-kβ signaling network (109). With p38 being one of renalase’s downstream signals (10, 11, 100), and NF-κβ being its transcription factor (100), it is possible that IL-6 and renalase share similar signaling circuits that may be triggered concurrently, presumably with context-dependent, thus opposing effects. Their opposite roles may be further rationalized by the evidence that renalase administration significantly decreases IL-6, being negatively associated with its expression (84). Furthermore, the NF-κβ and signal transducer and activator of transcription 3 (STAT3) work together to amplify the actions of IL-6, resulting in the production of a plethora of proinflammatory cytokines, including IL-6, and monocyte chemoattractant protein 1 (MCP-1). It is possible that the same pathways (NF-κβ and STAT3) induce renalase transcription in the same way that IL-6 most likely supervises, and even suppresses inflammation. This hypothesis provides a plausible explanation for renalase involvement in the context of cardiac fibroproliferative drive induced by IL-6 (110), particularly following the recognition that renalase’s anti-inflammatory traits are likely to be obtained, to some extent, by renalase’s suppression of macrophage-dependent IL-6 release (10). Moreover, a growing body of evidence indicates that the induced expression of IL-6 significantly contributes to the cardiac fibrotic process, through the activation of the PI3K/Akt/NF-kβ pathway (110), whereas the cytoprotective actions of renalase are evidenced to be mediated *via* PI3K/Akt and MAPK pathways, as discussed in detail previously. Moreover, the increased generation of ROS and IL-6 levels synergistically activate janus kinase (JAK)1/2-STAT1/3 signaling circuit, ultimately leading to activation of TGF-β1 profibrotic signals (111), whereas the initial neutralization of IL-6 attenuates the activation of cardiac fibroblasts, thus providing significant prevention of cardiac fibrosis (111, 112). The cardiac fibroblasts induced by macrophages’ stimulation also secrete IL-6, resulting in the synthesis and secretion of TGF-β1, eventually contributing to cardiac fibrosis (113). Another possibility that links the expression of renalase and IL-6 may be the STAT3-dependent stimulation, knowing that IL-6 confers a wide range of profibrogenic effects through the particular STAT3-dependent stimulation (that is under the supervision of renalase) of cardiac fibroblasts for collagen production (114), or induction of TGF-β1 (115). Indeed, a feedback loop of STAT3 with renalase has been demonstrated (100), whereby activated STAT3 boosts renalase expression, with renalase, in turn, regulating STAT3 function (10, 100). Regarding STAT3 itself, the latest consideration for its targeting during cardiac fibrosis progression has emerged to be the supervision and adjustment of its function, rather than large-scale perturbations (116), which seemingly may be renalase’s functional competence (10, 11, 100). However, renalase’s interaction with proinflammatory pathways necessitates far more investigation; thus, it would be interesting to investigate whether renalase modulates the detrimental cardiac profibrotic properties of TNF-α’ , and IL-6 and if so, to what extent.

Given its anti-inflammatory properties, it may be anticipated that renalase efficiently mitigates ROS production in various organ-damaged animal models (12–14, 18, 19, 44, 68, 84, 102, 117), warranting its primacy in becoming a marker for the severity of oxidative tissue injury, and for an assessment of the effects of applied antioxidant therapy (44). The discovery that some of renalase’s functions are dependent on its oxidase properties, and that renalase efficiently suppresses the generation of ROS, opens the door to its possible role in fibrotic myocardium. However, the lack of renalase is associated with a significant fall in the cellular nicotinamide adenine dinucleotide, the NAD^+^/NADH ratio, and decreased plasma NADH oxidase activity (118). These are among the reasons for aggravated myocardial injury, implying that renalase functions as a NADH oxidase, whereby the relationship between renalase and NAD^+^ is thoroughly discussed in the following subsection. Nevertheless, ROS represents a common link between cardiac injury and fibroproliferative response, operating as a harbinger of fibrosis (1, 26, 119), whereas it is broadly accepted that increased generation of ROS, by activating various signaling networks, represents one of the initial and essential mechanisms in the pathogenesis of cardiac fibrosis (1–3, 5, 6, 26, 119). Several lines of evidence indicate that ROS, derived from NADPH oxidase 4, induces collagen synthesis in cardiac fibroblasts (120, 121) throughout its signaling, particularly the activation of Akt/mTOR and NF-κβ cascade, whereas their suppression significantly reduces cardiac fibrosis (121). Moreover, the profibrotic properties of TGF-β1, including some other growth factors exerting similar fibroproliferative traits, are mediated by ROS (35, 74, 122), whereas their reciprocal regulation with TGF-β1 also exists, creating a vicious cycle for the progress of fibrosis (71). Furthermore, evidence suggests that ROS, primarily produced by nicotinamide adenine dinucleotide phosphate (NAD(P)H) oxidase, is likely responsible for TGF-β1-mediated myofibroblast differentiation, contributing to fibroblast differentiation into a myofibroblast phenotype (71, 122–124). Accordingly, pharmacological inhibition of cardiac oxidative stress results in a significant decrease of ECM proteins such as collagen I, collagen III, and α-SMA deposits, also associated with suppressed TGF-β signaling (125). Finally, ROS mediates TGF-β-induced EMT, *via* several signaling cascades, including Smad and Smad-independent (MAPKs and PI3K/Akt) (4, 7, 34, 126) pathways, whereas the Smad-independent network is, as already stated, under the supervision of renalase.

The cytoprotective potential of renalase was once again verified by its ability to favorably suppress the expression of monocyte chemoattractant protein (MCP)-1, chemokine nomenclature: C–C motif chemokine ligand 2/CCL2, and macrophage inflammatory protein (MIP)-1a, chemokine nomenclature: C–C motif chemokine ligand 3/CCL3, intercellular adhesion molecule (ICAM-1), all identified as markers of systemic inflammation, suggesting that renalase most likely rises to counteract the proinflammatory cascade, therefore exerting anti-inflammatory nature (12, 84, 106). In an injured heart, however, the most prominent chemokines for fibroblast progenitor chemotaxis activation, including macrophage recruitment and activity, that are of particular interest for the scope of this review, are evidenced to be MCP-1/CCL2 and MIP-1a/CCL3 (1, 26, 127–133). There is substantial evidence that the injured heart-induced mononuclear cell chemoattractants expression critically regulates the inflammatory response and induces fibrosis (129, 132), whereas these chemokines have a significant role in the long-term regulatory processes, such as fibroproliferation, by inducing macrophage differentiation and polarization (1, 26, 130). The observation that the lack of the renalase gene significantly increases MCP-1/CCL2 expression, and that renalase administration efficiently suppresses it, as evidenced *in vivo*, provides more layers of complexity to the biology of renalase and supports another scientifically plausible explanation for renalase’s anti-inflammatory efficiency within a clinical context. As demonstrated, injured heart overexpression of MCP-1/CCL2 induces myocardial IL-6 secretion, and subsequent activation of cardiac fibroblasts, contributing to a fibroproliferative response (133). Accordingly, the analysis of a significant positive correlation between increased circulating levels of MCP-1/CCL2 and circulating fibrocytes in cardiac fibrosis indicates that the pro-fibrotic role of MCP-1 includes, to some extent, the recruitment of myofibroblast progenitors to the site of the injury (3, 134, 135). Moreover, cardiac injury followed by significant and enduring activation of MCP-1 is seemingly followed by an increased expression of collagen I, α’ -SMA, CD34, and CD45, thereby myofibroblast activation (127, 136, 137). This action may be delayed by either genetic deletion of MCP-1 or injection of neutralizing anti-MCP-1 antibodies (136, 137), or perhaps some other newly identified molecule aiming to modulate its activation, presumably renalase, based on the *in vivo* evidence (12, 84, 106).

In the context of ongoing analysis into renalase’s anti-inflammatory nature, it deserves mentioning that deficiency of renalase, in the setting of acute kidney injury *in vivo*, results in significant neutrophil tissue accumulation, whereby pretreatment with renalase reduces the count of neutrophils infiltrating the tissue (106). Neutrophils are the first cells recruited to the site of cardiac injury, operating as a pro-inflammatory cell type, and according to current expertise, they significantly contribute to inappropriate fibrosis development (2, 3, 5–7, 29, 30, 138–140). Data to date shows that neutrophils, similar to macrophages, undergo polarization from pro-inflammatory (N1) cells to an anti-inflammatory subset (N2), whereas the N2 phenotype is obtained during the reparative phases of cardiac wounding to produce ECM proteins and contribute to scar formation (138–141). Moreover, the partnership of the neutrophil’s essential functions, such as degranulation and generation of ROS, when aggravated and unsupervised, may be detrimental, further contributing to the already injured cardiac issue (142). These data imply that neutrophils, as the predictors of cardiac remodeling and adverse clinical outcomes, allow them the advantage of being one of the targets for the full effectiveness of antifibrotic therapy (143). Another neutrophil response to cardiac injury is to activate the inflammasome, thus inciting chronic inflammation, apoptosis, and fibrosis (3) which hypothetically may be suppressed by renalase. For further clarification, the NLRP3 inflammasome, as aforementioned, represents an intracellular macromolecular structure that, after being triggered by perilous signals, releases predominantly IL-1β, to generate the inflammatory process (144–146), and as evidenced, its activation, and subsequent IL-1β production may be successfully hindered by renalase administration (10, 11, 13, 47, 67), and will be discussed hereafter. The extensively accumulated neutrophils contribute to cardiac fibrosis through the production of extracellular traps (NETs), whereby fibroblasts exposed to NETs achieve competence to proliferate, transdifferentiate, and produce collagen, while at the same time inducing macrophage polarization toward a pro-fibrotic subset (145, 147). The short life span of neutrophils, being evolutionary conservative, aims to prohibit chronic inflammation and excessive collagen deposition, yet cardiac fibrosis progression, provides a proof of principle that their supervised accumulation, perhaps by renalase, may provide favorable feedback in the course of cardiac fibrosis initiation and evolution. Finally, renalase administration increases antiapoptotic factors, such as Bcl-2, and the prevention of caspase-3 activation (12–14, 102, 117). A schematic view of the renalase-targeted cellular effectors and pathways in the TGF-β-induced cardiac fibrosis is presented in Figures 2, 3, and accordingly summarized in Table 1.

Taken together, mounting research (11, 15, 17, 18, 44, 45, 47, 48, 68, 84, 102, 105, 117) adds substantial evidence that renalase confers cytoprotection, by exerting anti-inflammatory, antioxidant, and anti-apoptotic effects, providing a hypothesis of a single molecule for targeting various pathways. Given that tissue safety is impeded by the silencing of the renalase gene, recombinant renalase administration, accordingly, generates robust protection, and preserves the phenotypes of injured cells, including cardiomyocytes, and upholds the possibility that renalase administration may be relevant in cardiac fibroproliferation.

## The Renalase-Dependent Signaling of Macrophages Within the Context of Cardiac Fibrosis

The proposed relationship between renalase and macrophages deserves to be discussed as a separate section, owing to a few noteworthy macrophage responses incited by renalase, particularly in light of a significant role for monocytes and macrophages as regulators of cardiac fibrosis (148–155). To date, there is substantial evidence that renalase *in vivo* provides several beneficial effects, such as the hindrance of total macrophage accumulation (10–13, 19, 20, 47, 67, 84, 102), specifically the inflammatory phenotype (20, 84), polarizing them toward the M2 phenotype (84), and blocking inflammasome activation, and IL-1β secretion (10, 47, 67). These observations uphold the need for more meticulous research regarding the hypothetical relationship of renalase to macrophage triggering, and the pathways involved therein.

However, apart from a simple inhibition of their accumulation, renalase evidently interferes with macrophage phenoshifting, polarizing them toward the M2-like subset. As noted in the previous sections, macrophage shift from M1 to a M2-like phenotype presumably has a causative role in the context of heart protection (5, 6, 27, 36, 150–155), presuming that the specific modulation of this action may be highly beneficial for withholding exaggerated fibroproliferative responses. Moreover, possible disorganization between these macrophages’ profiles, according to some authors, represents, at least partially, an underlying mechanism of overwhelmed myocardial inflammation and subsequent cardiac fibrosis (1–3, 5, 6, 152–155). To accomplish appropriate evolution from the stage of cardiac tissue repair to a regenerative point, cardiac macrophages most likely have to be supervised, and fine-tuned between pro-fibrotic and anti-fibrotic traits (6, 7, 151, 153), particularly since macrophages are cells characterized by high plasticity (3, 5, 6, 31, 32, 148–155), whose functions may be context-dependent (steady-state or injury). In line with that, it is confirmed that during the proliferative phase, a macrophage’s modulation aims to boost the M2-phenotype, and deplete M1-macrophages, and results in a significant lessening of interstitial cardiac fibrosis (3, 5, 6, 154). The specific mediators, or supervisors of this particular macrophage drive are, however, still not widely recognized (6, 31, 32, 151, 155), and this action, perhaps, may be regulated by renalase administration. However, data from several experimental studies supports the hypothesis that macrophages may seemingly be targeted by renalase (Figure 2), to obtain the desired phenotype, and subsequent appropriate healing response. Evidence suggests that the absence of the renalase gene in experimental mice following diverse types of injuries, in addition to significantly upregulated TNF-α’ , MCP-1, and MIP-2, is followed by significant renal (11–13, 84, 102), liver (19), and pancreatic macrophage accumulation (47). Moreover, the most recent data (20) shows that genetic deletion of renalase results in significantly more severe cisplatin-induced chronic kidney disease, and that renalase agonist peptide (RP81) supplementation, *via* mesoscale nanoparticles, decreases inflammatory cytokines plasma levels, and preserves epithelial components of the nephron, and the vasculature, and suppresses inflammatory macrophages and myofibroblasts. The observation that macrophages express the extracellular renalase-targeting PMCA4 receptor (47, 156), additionally spotlights the hypothesis that renalase is involved in the appropriate stimuli presented to macrophages, and may be essentially involved in their subsequent phenotype adjustment. It also deserves mentioning that renalase, and its receptor, PMCA4b, are expressed in all layers of the placenta, implying a potential role for renalase in placental development and function (25). Renalase’s activation of these particular receptors proves valuable in pancreatic tissue protection during *in vivo* induced injury (47), whereas the scarcity of renalase is highly associated with profound tissue deterioration, since renalase knockout mice experience significantly more severe macrophage infiltration (47). It may be feasible that renalase regulates the influx of pro-inflammatory macrophages (M1) during the initial phase of an injury, and perhaps even conducts their later transition into a reparative M2-like subset, as renalase was also highly associated with macrophages presenting with an anti-inflammatory M2 phenotype (47, 84). Such evidence favors renalase’s possible role in mediating, and controlling macrophage phenotype polarization. Nevertheless, cardiac macrophage proliferation is promoted *via* the activation of MAPKs signaling pathways (150, 157), the same downstream signals mediated by renalase through its PMC4b receptor (10, 11, 16, 47, 51, 67, 100). Moreover, renalase gene deletion results in a significant increase in MCP-1, and MIP-2 expression, followed by massive macrophage infiltration, whereas renalase administration ameliorates the damaged phenotype, and reduces macrophage accumulation (12, 13, 47, 84, 102). In this manner, the inverse correlation of MCP-1 and MCP-2 upregulation and renalase expression suggests that renalase influences the cells of interest, seemingly macrophages (47), whereas the major observation is that renalase alters the M1/M2 phenotype (84). Renalase administration, however, significantly eliminates the accumulation of total macrophages (CD68), and M1 (CD86) linage, followed by an increase in the M2-like (CD 163) phenotype. It deserves emphasis that the M2 phenotype, found in tumors, was also highly associated with renalase expression, once again indicating that renalase’s signals may be aimed toward the acquisition of an anti-inflammatory phenotype (10, 11, 20, 47, 67, 84, 102). The related research on renalase’s effects on liver injury (19) reveals that the lack of the renalase gene favors, apart from oxidative stress promotion, significant macrophage accumulation, evidenced by the increased expression of the adhesion G-coupled receptor E1 (Adgre1), a mature macrophage marker. Although still scarce, the data viewed on renalase’s traits supports the hypothesis that this molecule, by tailoring profibrotic and anti-fibrotic macrophage phenotypes, may have a few possible cardioprotective implications in the evolution of cardiac fibrosis.

In accordance with this possibility, further notable evidence of renalase’s ability to affect macrophages *via* hindering their inflammasome activity when activated (10, 11, 13, 47) may also be argued in the context of cardiac fibrosis. According to a growing body of evidence (37, 38, 75, 76, 144, 146, 158–164), a greater assembly of inflammasomes (particularly NLRP3), and their persistent activation, have been shown to significantly contribute to cardiac fibrosis under various pathologic conditions, and their expression is associated with an increased cardiovascular risk and mortality (76). The activation of the inflammasome first endeavors to induce myofibroblast-mediated wound repair (75, 158–163). Its sustained activity, however, maintains chronic cardiac inflammation, and subsequent fibrosis progression, whereas *via* generation of IL-1β, IL-18, and ROS, it provides increased TGF-β expression. Accordingly, the particular inflammasome-targeted therapy demonstrates some beneficial effects on cardiac fibrosis (75), leading to the introduction of previously unrecognized molecules (75, 158–160), that may effectively suppress NLRP3 inflammasome activity. The described pathophysiology is worthy of comparison, and essentially corresponds to the proposed renalase’s ability to abort the activation of the inflammasome (as experimentally observed). However, the precise details of these processes within discussed mechanisms are yet to be clarified. With reference to the aforesaid data, an understanding that the process of epithelial to mesenchymal transition also requires the inflammasome complex activation, and that EMT does not occur when the inflammasome is silenced (164), opens another door of possibility for renalase’s purposeful scrutiny. Additionally, the epithelial to mesenchymal transition process, which contributes to the pool of myofibroblast in cardiac tissue as already stated, has been reported to be, to some extent, suppressed by renalase (68, 69). There could be a possibility that renalase, *via* silencing the inflammasome, indirectly adjusts EMT activation, thus facilitating cardiovascular healing. An illustration of renalase-targeted cellular effectors, including macrophages, is presented in Figure 2, and accordingly summarized in Table 1.

Yet another challenge to address within the context of cardiac fibrosis and renalase’s possible role therein, would be to define the proper cytoprotective mechanism elicited by renalase triggering its receptor (10, 11, 16, 47, 51, 67, 100). Whether the renalase PMCA4-activated signals initially affect calcium efflux, or synergistically engage various renalase-dependent downstream signaling pathways, namely PI3K/Akt, ERK 1/2, p38, B cell lymphoma 2, and cyclic adenosine monophosphate (cAMP) (10, 11, 13, 16, 165) remains to be elucidated. A few premises may be offered in order to understand renalase’s possible activity with reference to the PMCA4 receptor. A likely target for cardiac fibrosis regulation by controlling the local calcium and protein-to-protein interactions has emerged to be the PMCA4 (166, 167), which is documented to be the renalase-targeting receptor. The PMCA4 also regulates the expression of major signaling molecules in cardiac fibroblasts, whereby diverse pharmacological modulation in the expression of fibroblasts’ PMCA4 receptors hinders induced cardiac fibrosis, and even manages to suppress, and even reverse these processes (167). Moreover, isolated cardiac fibroblasts express their biomarkers (α-SMA, collagen type I, and fibronectin) in a manner-dependent presence of extracellular calcium (Ca^2+^), implying Ca^2+^-transporting mechanisms (perhaps renalase-triggering PMCA4) to be a potential therapeutic vehicle in cardiac fibrosis (167, 168). The agreed consensus suggests that cAMP-regulated signaling pathways (another signal mediated by renalase’s triggering of the PMCA4 receptor (10), modulate fibrotic response in injured or stressed myocardium by affecting cardiac fibroblast function, whereas diverse modulators of that signaling may be essentially involved in the regulation of cardiac fibrosis mechanisms (169). For instance, cAMP triggering enhances several pro-fibrotic pathways, such as PI3K/Akt, which inhibit TGFβ-Smad2/3 and p38 signaling (169). Indeed, the inhibitory effects of cAMP regulators on collagen synthesis, and α-SMA expression have been demonstrated to require the activation of PI3K/Akt (169). PMCA4 triggering, including renalase, mediates PI3K/Akt-dependent mechanisms, probably with the primary intention of maintaining ATP levels, and presumably affecting some other PI3K/Akt-independent pathways (47). A tempting, but so far only theoretical approach, that renalase may enhance the activity of anti-fibrotic cAMP, and inhibit signals through pro-fibrotic cAMP complexes, justifies future intense analysis of this possible action. Finally, genetic or pharmacological inhibition of PMCA4b suppresses renalase-induced signaling, including its cytoprotective behavior, thereby supporting the importance of PMCA4b in receptor-mediated extracellular renalase function (165). The discovery that intracellular renalase levels vary in the same direction as extracellular renalase (67, 165), and presumably play some metabolic roles, such as post-translational modifications and enzyme activity (55, 165), may be of particular interest, especially given that macrophage metabolic shift is required to maintain pro- and anti-inflammatory processes (6, 152). The metabolic shifts of macrophages are tightly regulated by specific intracellular signals (NF-κβ and HIF-1α’ ), a few of which share transcription factors with renalase (10, 100). Such a hypothesis lacks experimental evidence, and is beyond the scope of this review.

Based on the evidence, it is plausible that renalase’s triggering of PMCA4 receptors regulates pathophysiological responses such as macrophage polarization (M1/M2), Ca^2+^ handling, cAMP signalization, and PI3K/Akt-dependent mechanisms. Whether renalase may fine-tune the stimuli for macrophages to acquire a specific functional profile (pro- and antifibrotic) in the cardiac tissue, in the same manner as demonstrated in the kidney, and pancreatic tissue, is an intriguing goal for future research. As compelling as this theory is, however, it is still highly hypothetical and requires considerably more in-depth profiling to identify the proper renalase to macrophage signals that supervise these interactions after cardiac injury.

## The Regulatory Interplay Between Renalase and Sirtuins in the Context of Cardiac Fibrosis

In the light of renalase’s pathophysiological relevance, apart from its anti-inflammatory actions and cytokine nature, renalase’s enzymatic behavior should be considered. Although renalase is initially identified as a molecule that regulates catecholamine levels (42, 43, 53), the focus of subsequent research shifts to its oxidative metabolism of nicotinamide adenine dinucleotide (NAD(P)H) (10, 54, 55). Nonetheless, evidence suggests that renalase acts as an NADH oxidase (10, 13, 54, 55, 61, 118), specifically oxidizing and epimerizing α’ -NAD(P)H molecules (54, 55, 170). Acknowledging this has two powerful biological implications. First, renalase may preferentially hinder the accumulation of unstable molecules, acting as a “scavenger” (54), as already argued, and secondly, it converts 2 or 6 NADH, which are metabolically inert, to active 4-NAD^+^, whose metabolism is closely linked to the cellular energy metabolism (118). In line with that, the renalase knockout mice experience more severe cardiac ischemic lesion (e.g., the degree of the infarction area), whereby recombinant renalase application rescues the overall myocardial phenotype, including a significant improvement in heart function (118). This may be revealed due to the discovery that the absence of the renalase gene results in a significant decrease in plasma NADH oxidase activity, as well as in the NAD^+^/NADH ratio within cells, most likely due to its function as an oxidase/anomerase, that uses molecular oxygen to convert α’ -NAD(p)H to β-NAD^+^ (54). The concept that renalase is likely to provide sustained regulation of NAD^+^ levels (10, 54, 55, 118) has escalated the focus of its research to a whole new stage (14, 117), owing to the clear association of NAD^+^ with the sirtuin family, whose functions are NAD^+^-dependent. To clarify this hypothesis, it should be noted that elevated levels of NAD^+^ positively upgrade the actions of sirtuins (SIRTs), which are specifically involved in cell survival and metabolic homeostasis (171–174). However, sirtuins are identified to be the NAD^+^-dependent type III deacetylases that may be collectively activated by increasing their co-substrate NAD^+^ (173). This knowledge, in line with renalase’s capability to modulate the pool of NAD^+^, in the experimental setting, provides the proof of principle that renalase may be indirectly implicated in the modulation of sirtuin activities. This novel relationship may be of particular interest regarding the scope of this review, since there is ample current evidence that implies sirtuins significance in myocardial fibrotic response, and their cardioprotective effects in experimental settings (173–195). However, this is the first review that scrutinizes renalase’s activity through its relationship with sirtuins.

Sirtuins 1–7 are a highly conserved group of NAD^+^-dependent deacetylating enzymes, allocated to various cellular sections (nucleus, mitochondria and cytosol), and target many cell proteins, intending to provide protection against aging, metabolic diseases, hypoxia, and chronic inflammation (171–182). Sirtuin (SIRT) 1, the best-characterized member of the sirtuin family, is located in the nucleus and cytosol, providing its actions by multiple mechanisms: coordinating oxidative energy metabolism and inflammatory responses through the interaction with NF-kβ (183), impeding hypoxia-induced apoptosis (*via* inositol-requiring enzyme-1α signaling), and subsequent cardiomyocyte protection from hypoxic stress, and autophagy modulation (*via* AMPK upregulation (172, 175). In general, SIRT1 is observed as a principal stress adaptor, regulating the activity of NOS, p53, angiotensin II type 1 receptor, and forkhead box O (FOXO) (174–176), providing protection from apoptosis, aging, senescence, and chronic inflammation (171–176, 179, 180, 182–187). Additionally, recent research has confirmed the cardioprotective traits of SIRT1 in the context of cardiac fibroproliferative response *via* diverse signaling networks. For instance, SIRT1, when upregulated, may prevent, or at least delay senescence, and ameliorate the function of aged mesenchymal stem cells, thereby suppressing cardiac fibroblast activation (175). Sirtuin 1 may particularly target pro-fibrotic pathways, such as Smad 2/3, so pharmacological activation of SIRT1 may be a new strategy to attenuate, and even reverse cardiac fibrosis *via* Smad activity modification (179). Similarly, in the experimental model of cardiac fibrosis, the upregulation of SIRT1 results in TGF-β, and pSMAD3/SMAD3 suppression, followed by significantly reduced fibroblast activation (180). Furthermore, SIRT1 has the potential to reduce macrophage-based NF-κβ activation (183), resulting in significant anti-inflammation that slows fibrosis progression, and regulates p53 activity in cardiomyocytes (173). Sirtuin 1 activation also improves mitochondrial function, reduces oxidative stress and fibrosis, silences proinflammatory pathways (184), and prevents profibrotic phenotypic alterations in the myocardium by inhibiting NF-κβ and MMP9 transcriptional activation (185). Furthermore, SIRT1 upregulation allows for the deactylation of NF-κβ (183), which aids in the management of inflammation, oxidative stress, and apoptosis (186), thus interfering with the MAPKs pathways, *via* Akt/ASK1 signaling, by reducing p38 and JNK phosphorylation, and increasing ERK 1/2 phosphorylation (187). These actions regulate fibroblast activation and myocardial fibrosis (187). Sirtuin 1 may negatively regulate cardiac fibroblast transdifferentiation, and *vice versa*; this, along with its downregulation, may enhance cardiac fibroblast proliferation, and upregulate the expression of the fibrosis-related genes *via* NF-κβ signaling.

Comparatively, abundant evidence suggests that the downregulation of mitochondrial sirtuin 3 (SIRT3) has a causative role in a number of cardiac pathologies, including the development of cardiac fibrosis and adverse heart remodeling (173, 177, 178, 181, 182, 188–195). In particular, SIRT3 deficiency results in exacerbated cardiac hypertrophy and subsequent cardiac fibrosis, whereas cardiac hypertrophy, followed by fibrosis, can be inhibited by the upregulation or activation of SIRT3 (188), presuming that SIRT3 mitigates cardiac fibroproliferation (182, 188). Similarly, SIRT3 deficiency promotes age-related cardiac fibrosis, presumably due to impaired deacetylation and inhibition of glycogen synthase kinase 3b (GSK3b), which leads to increased TGF-β expression, whereas SIRT3 activation of GSK3β prevents the TGF-β/Smad3-mediated cardiac fibrotic response (181, 182, 188, 189). Furthermore, the activation of SIRT3 by resveratrol ameliorates cardiac fibrosis and improves cardiac function *via* the TGF-β/Smad3 pathway (190). The depletion of SIRT3 enhances angiotensin II-induced NADPH oxidase-derived ROS formation, followed by the upregulation of TGF-β, suggesting that cardiac fibrosis is, to some extent, provoked by pathways involving SIRT3-mediated pericyte-myofibroblast/fibroblast transition and the ROS-TGF-β pathway (177). Moreover, SIRT3 modulates proinflammatory and profibrotic signals in cardiomyocytes *via* FOS/AP-1 pathways, whereas the SIRT3 knockout mice experienced significant myocardial fibrosis associated with increased activity of AP-1 transcriptional activity (178), implying that SIRT3 activation is a potential vehicle for treating cardiac fibrosis. The lack of SIRT3, *in vitro*, allows resident cardiac fibroblasts to easily transdifferentiate into myofibroblasts, producing more fibrotic mediators, including TGF-β, aiming to maintain cardiac fibroproliferation. These actions are accomplished *via* the STAT3-NFATc2 (191, 192), and β-catenin/PPAR-γ signaling pathways (191, 193). Finally, mice that lack SIRT3 manifest interstitial fibrosis by 8 weeks of age (194), whereas the overexpression of cardiac-specific SIRT3 ameliorates doxorubicin-induced cardiac fibrosis, and fetal gene expression (194). Several mechanisms may contribute to the beneficial effects of SIRT3. However, concerning this review, the emphasis should be on the potential of SIRT3 to alleviate the ROS-sensitive MAPK/ERK 1/2 and PI3K/Akt pathways (191), particularly with the knowledge that this signaling network has been identified to play a key role in the development of cardiac fibrosis (7, 34). Nevertheless, increasing evidence shows that pharmaceutical avenues by supplementing NAD^+^ boosters to obtain tissue-specific modulation of sirtuins may be harnessed in the context of cardiac inflammation and fibrosis (173, 180–182, 191, 194–198).

With reference to the aforementioned, current expertise on renalase’s pathophysiology permits a scientific rationale for the hypothesis that renalase administration may significantly contribute to the cellular NAD^+^ pool, thereby providing the recovery of NAD^+^ content (Figure 3), and hence enabling sirtuins activation (14, 117). It is established that renalase significantly upregulates SIRT1 activity for the alleviation of detrimental ischemia/reperfusion events by mitigating oxidative stress and mitochondrial damage in liver injury (117). The study, however, provides evidence that downregulation of SIRT1 expression and activity is significantly associated with the lack of the renalase gene and downregulated NAD^+^ levels. Additionally, the upregulation of NAD^+^, and subsequent activation of SIRT1, provided by renalase administration, remarkably deplete the acetylation level of p53, negatively regulate Bax expression, and enhance Bcl-2 expression (117), further confirming renalase’s protective role. Apart from its antioxidant traits, which have been discussed, this particular study shows that renalase administration rescues the mitochondrial network morphology, evidenced by the significant suppression of mitochondrial fission-related protein (Drp1) following recombinant renalase application. The related study (14) reveals (*in vitro* and *in vivo*) that renalase pretreatment significantly suppresses cisplatin-induced upregulation of cleaved caspase-3 and downregulation of Bcl-2, therefore preventing apoptosis and decreasing mitochondrial ROS production. The realization that is reinforced, however, is that recombinant renalase, following cisplatin treatment, significantly recovers the expression of SIRT3, prevents its downregulation, and reduces mitochondrial fragmentation. Taken together, renalase, by upregulation of SIRT3 levels, most likely exerts protection in a SIRT3-dependent manner. A schematic view of the interplay between renalase and sirtuins is given in Figure 3, and summarized in Table 1.

Such promising results, admittedly based on relatively scant data, point out an intriguing role for renalase, not just to indirectly target and upregulate sirtuins (1 and 3), but also to operate as a “guardian of the mitochondria”. This hypothesis offers the scientific community a new area of research to be addressed by further scrutiny: whether renalase could maintain the activity of sirtuins and mitochondrial dynamics during the progression of cardiac fibrosis (39, 173, 174, 199).

## Conclusion

Cardiac fibrosis represents a redundant accumulation of ECM resulting from a cascade of pathophysiological events involved in an ineffective healing response, that eventually leads to heart failure. Even though outstanding advancements in the last decade have been made in order to understand cardiac fibrosis pathogenesis, its efficient therapeutic entities, albeit impatiently awaited, remain a major challenge. Cardiac fibrosis does not represent an isolated phenomenon, but rather simultaneous or successive responses to various signals directed toward cell types involved in the fibrotic feedback. Consequently, the most effective strategy will likely have to incorporate the specific targeting of the diverse cells, pathways, and their cross-talk in the pathogenesis of cardiac fibroproliferation.

Due to the intense scrutiny of renalase’s pathophysiology in recent years, its resulting molecular signature has gained attention for its relevance in tissue protection, and fibrosis alleviation. Owing to its functional diversity, evidenced *in vitro* and *in vivo*, it may be possible that renalase provides an appropriate tissue response, presumably by fine-tuning and adjusting anti-inflammatory and pro-fibrotic pathways. Effective antifibrotic therapy may seek to exploit renalase’s compound effects such as: lessening of the inflammatory cell infiltrate (neutrophils and macrophages), and macrophage polarization, a decrease in the proinflammatory cytokine/chemokine/reactive species/growth factor release (TNF-α, IL-6, MCP-1, MIP-2, ROS, TGF-β1), an increase in antiapoptotic factors (Bcl2), and prevention of caspase activation, inflammasome silencing, sirtuins (1 and 3) activation, and mitochondrial protection, suppression of epithelial to mesenchymal transition, and a decrease in the profibrotic markers expression (α’ -SMA, collagen I, and III, TIMP-1, and fibronectin). Perhaps the most captivating feature of renalase that provides a scientific rationale for its further scrutiny in the field of cardiac fibrosis is renalase’s interference with MAPKs signaling network, most likely as a coordinator of pro-fibrotic signals.

Although substantial progress has been made, indicating renalase’s therapeutic promise, more extensive and profound experimental work is required to resolve the accurate underlying mechanisms of renalase, concerning myocardial diseases, and cardiac fibrosis, before any potential translation to clinical investigation. This review should, however, help in creating a better knowledge base, and a framework for further investigation. Finally, owing to the emerging concepts, the efforts of the entire scientific community should be concerted to set the stage for myocardial fibrosis research at an advanced level, searching for novel therapeutic avenues to prevent fibrosis in failing hearts.

## Author Contributions

DS conceptualized, edited the manuscript, searched the literature, and together with the coauthors wrote the manuscript. All authors read and provided critical review of the article, and approved the final version for publication.

## Conflict of Interest

The authors declare that the research was conducted in the absence of any commercial or financial relationships that could be construed as a potential conflict of interest.

## Publisher’s Note

All claims expressed in this article are solely those of the authors and do not necessarily represent those of their affiliated organizations, or those of the publisher, the editors and the reviewers. Any product that may be evaluated in this article, or claim that may be made by its manufacturer, is not guaranteed or endorsed by the publisher.
